# The prevalence of primary headache and its association with socioeconomic factors among school children in Lithuania

**DOI:** 10.1186/1129-2377-14-S1-P3

**Published:** 2013-02-21

**Authors:** A Januskeviciene, A Zaborskis, E Vaitkaitiene

**Affiliations:** 1 Lithuanian University of Health Science, Lithuania, Institute for Biomedical Research of Kaunas University of Health Science , 4, Eiveniu str., Kaunas, LT-50009, Lithuania

## Objective

The aim of this study was to estimate the prevalence of headache among children in Lithuania in 2010 and describe the association between headache and socio-economic factors. This study was a part of the Cross-National Survey on Health Behaviour in School-aged Children – World Health Organization Collaborative Study (HBSC).

## Methods

The research was carried out according to the methodology of the HBSC study using the anonymous standardized questionnaire. In total, 5,323 students (2, 740 (51.5 %) boys and 2,583 (48.5 %) girls aged 11, 13 and 15 years from Lithuania were surveyed in the 2010 school-year.

## Results

The total prevalence of frequent headache (at least once a week) among children 11, 13 and 15 years of age was 33,7% 31,9% and 34,4% , respectively. Pain was most frequent among older girls (13 and 15 years of age ) (χ2 = 43,529 ; df = 2; p< 0.005). The headache prevalence was slightly higher in low-income families and divorsed or one parent families compared to those of high status and two parent families. Multivariate analysis revealed that having a good relationship with parents and peers, associated with less headache cases.

## Conclusion

Headache is more common among children with lower socioeconomic groups. Social causation can play a role in the pathogenesis of headache and should be considered by health promoters.
